# *Haemophilus haemolyticus* Interaction with Host Cells Is Different to Nontypeable *Haemophilus influenzae* and Prevents NTHi Association with Epithelial Cells

**DOI:** 10.3389/fcimb.2016.00050

**Published:** 2016-05-09

**Authors:** Janessa L. Pickering, Amy Prosser, Karli J. Corscadden, Camilla de Gier, Peter C. Richmond, Guicheng Zhang, Ruth B. Thornton, Lea-Ann S. Kirkham

**Affiliations:** ^1^Wesfarmers Centre of Vaccines and Infectious Diseases, Telethon Kids Institute, The University of Western AustraliaPerth, WA, Australia; ^2^School of Paediatrics and Child Health, The University of Western AustraliaPerth, WA, Australia; ^3^Department of Paediatrics, Princess Margaret Hospital for ChildrenPerth, WA, Australia; ^4^School of Public Health, Curtin UniversityPerth, WA, Australia

**Keywords:** colonization, *Haemophilus haemolyticus*, host epithelium, inflammatory mediators, *in vitro*, NTHi, PBMCs

## Abstract

Nontypeable *Haemophilus influenzae* (NTHi) is an opportunistic pathogen that resides in the upper respiratory tract and contributes to a significant burden of respiratory related diseases in children and adults. *Haemophilus haemolyticus* is a respiratory tract commensal that can be misidentified as NTHi due to high levels of genetic relatedness. There are reports of invasive disease from *H. haemolyticus*, which further blurs the species boundary with NTHi. To investigate differences in pathogenicity between these species, we optimized an *in vitro* epithelial cell model to compare the interaction of 10 *H. haemolyticus* strains with 4 NTHi and 4 *H. influenzae*-like haemophili. There was inter- and intra-species variability but overall, *H. haemolyticus* had reduced capacity to attach to and invade nasopharyngeal and bronchoalveolar epithelial cell lines (D562 and A549) within 3 h when compared with NTHi. *H. haemolyticus* was cytotoxic to both cell lines at 24 h, whereas NTHi was not. Nasopharyngeal epithelium challenged with some *H. haemolyticus* strains released high levels of inflammatory mediators IL-6 and IL-8, whereas NTHi did not elicit an inflammatory response despite higher levels of cell association and invasion. Furthermore, peripheral blood mononuclear cells stimulated with *H. haemolyticus* or NTHi released similar and high levels of IL-6, IL-8, IL-10, IL-1β, and TNFα when compared with unstimulated cells but only NTHi elicited an IFNγ response. Due to the relatedness of *H. haemolyticus* and NTHi, we hypothesized that *H. haemolyticus* may compete with NTHi for colonization of the respiratory tract. We observed that *in vitro* pre-treatment of epithelial cells with *H. haemolyticus* significantly reduced NTHi attachment, suggesting interference or competition between the two species is possible and warrants further investigation. In conclusion, *H. haemolyticus* interacts differently with host cells compared to NTHi, with different immunostimulatory and cytotoxic properties. This study provides an *in vitro* model for further investigation into the pathogenesis of Haemophilus species and the foundation for exploring whether *H. haemolyticus* can be used to prevent NTHi disease.

## Introduction

*Haemophilus haemolyticus* is a respiratory tract commensal that is closely related to the opportunistic pathogen nontypeable *Haemophilus influenzae* (NTHi). NTHi is a major cause of otitis media (OM) in children (Murphy et al., 2009b; Wiertsema et al., 2011) and exacerbations of chronic obstructive pulmonary disease (COPD) in adults (Thanavala and Lugade, 2011; Alikhan and Lee, 2014). Additionally, NTHi causes sinusitis, conjunctivitis, pneumonia, bacteraemia and meningitis (Shann et al., 1984; Dworkin et al., 2007; Cripps, 2010; Laupland et al., 2011; van Wessel et al., 2011). The burden of invasive disease due to NTHi is steadily increasing, particularly in infants and the elderly (Laupland et al., 2011). There is added concern due to the emergence of antibiotic resistance within the species (Van Eldere et al., 2014). Although *H. haemolyticus* is generally considered a commensal, there are occasional reports of isolation of this bacterium from normally sterile sites (Anderson et al., 2012; Morton et al., 2012; Hariadi et al., 2015). The distinction of *H. haemolyticus* as a true commensal is complicated by the fact that *H. haemolyticus* can be misidentified as NTHi using routine microbiological tests (reviewed in Pickering et al., 2014b). Whilst in-depth investigations into the genetics of *H. haemolyticus* and NTHi have been conducted in order to develop discriminatory tests to distinguish these closely related species (McCrea et al., 2008, 2010a; Sandstedt et al., 2008; Norskov-Lauritsen, 2011; Binks et al., 2012; Pickering et al., 2014b), the interaction of *H. haemolyticus* with host cells has not been previously investigated.

Colonization of the human upper respiratory tract with NTHi precedes infection and studies have shown an association between a high density of NTHi carriage and subsequent development of OM (Smith-Vaughan et al., 2006, 2013). Although the progression from NTHi colonization to disease is not entirely understood, *in vitro* and *in vivo* studies have revealed that NTHi can persist in biofilms and/or intracellularly within the respiratory tract (Murphy et al., 2009a; Clementi and Murphy, 2011; Novotny et al., 2013; Jalalvand and Riesbeck, 2014). This makes NTHi respiratory infections such as OM difficult to treat with antibiotics (Dagan, 2000). Current preventative strategies against development of NTHi disease include the licensed pneumococcal conjugate vaccine (PHID-CV) that incorporates the NTHi lipoprotein Protein D (Prymula and Schuerman, 2009). PHiD-CV does not prevent NTHi colonization (van den Bergh et al., 2013; Hammitt et al., 2014; Feazel et al., 2015) and the impact of this vaccine on NTHi OM has been variable (Prymula et al., 2011; van den Bergh et al., 2013; Tregnaghi et al., 2014; Leach et al., 2015). Additionally, clinical Protein D-negative NTHi strains have been identified (Smith-Vaughan et al., 2014) highlighting the potential for PHID-CV evasion by NTHi. Indeed, sub-unit vaccines against NTHi are limited given the considerable antigenic variation within NTHi (Cripps et al., 2002; Price et al., 2015). A Cochrane review of six clinical trials with an oral whole-cell killed NTHi vaccine developed to reduce acute exacerbations in COPD patients revealed mixed results and studies that did show a benefit to COPD patients had too small numbers to warrant widespread vaccination (Teo et al., 2014).

An alternative approach to vaccination is the use of probiotic therapies with the potential to modulate the host microbiome and prevent infection. Data from trials of probiotics to prevent OM are conflicting with some studies showing protection (Skovbjerg et al., 2009; Lehtoranta et al., 2012; Di Pierro et al., 2014) and others demonstrating a lesser or no impact (Taipale et al., 2011; Cohen et al., 2013). To our knowledge, no studies have directly addressed the prevention of NTHi colonization with a commensal or probiotic species. We propose that *H. haemolyticus* may interfere with NTHi colonization of the respiratory tract and could therefore be exploited as a probiotic to reduce the burden of NTHi disease. This is not only due to the fact that both species colonize the respiratory tract and are highly related at a genomic level (Price et al., 2015) but also that *H. haemolyticus* is less often isolated from populations with high rates of NTHi carriage and disease (Fenger et al., 2012; Pickering et al., 2014c; Aho et al., unpublished). In order to explore the potential therapeutic use of *H. haemolyticus*, investigations into the interaction of *H. haemolyticus* with the host were required.

In this study, we used an established *in vitro* model of NTHi infection (Swords et al., 2000) to characterize *H. haemolyticus* association and invasion of human respiratory epithelial cells. As *H. haemolyticus* is generally considered to be a commensal, we hypothesized that nasopharyngeal *H. haemolyticus* isolates would be able to colonize host epithelial cells but not necessarily invade them like NTHi. To take into consideration the high level of strain variation, we compared host cell association and invasion of 10 *H. haemolyticus* isolates (including four naturally occurring Protein D-negative isolates and an invasive *H. haemolyticus* isolate) with four NTHi strains. We also assessed four representative isolates from a clade of “fuzzy” haemophili that have recently been differentiated from NTHi and *H. haemolyticus* by whole genome sequencing and are referred to as “*H. influenzae*-like haemophili” (Price et al., 2015). Inflammatory responses from epithelial cells challenged with these strains were evaluated and further assessed using peripheral blood mononuclear cells (PBMCs). Finally, we investigated whether pre-treatment of epithelial cells with *H. haemolyticus* could block NTHi attachment to cells to determine whether *H. haemolyticus* can inhibit NTHi colonization. This is the first study to assess *in vitro* interactions of *H. haemolyticus* with host cells. This study provides *in vitro* models to further investigate the pathogenesis of haemophili, the biological role of *H. haemolyticus*, and to determine whether *H. haemolyticus* may be used to prevent NTHi colonization and therefore disease.

## Materials and methods

### Microbial strains and growth conditions

The isolates assessed in this study were first described in four previous studies (Bakaletz et al., 1988; Nizet et al., 1996; Wiertsema et al., 2011; Morton et al., 2012) and are detailed in Table 1. All isolates were X and V factor dependent and were identified as either NTHi*, H. haemolyticus* or *H. influenzae*-like haemophili by *hpd*#3 PCR (Wang et al., 2011), HRM *hpd* PCR (Pickering et al., 2014a), *fuc*P PCR, or whole genome sequencing (Price et al., 2015).

**Table 1:** **Haemophilus isolates assessed in this study**.

**Isolate ID**	**Site of isolation**	**Origin (study reference)**	**PCR^b^**
**NTHi**			
86-028NP	Nasopharynx	U.S.A.; Bakaletz et al., 1988	*hpd#3^+^, fucP^+^*
R2866	Blood	U.S.A.; Nizet et al., 1996	*hpd#3^+^, fucP^+^*
H76	Middle ear	W.A.; Wiertsema et al., 2011	*hpd#3^+^, fucP^+^*
H94	Nasopharynx^a^	W.A.; Wiertsema et al., 2011	*hpd#3^+^, fucP^+^*
***H. haemolyticus***			
Hh33390	Sputum	ATCC® 33390™	*hpd#3^−^, fucP^−^*
HI2028	Blood	U.S.A.; Morton et al., 2012	*hpd#3^−^, fucP^−^*
H12	Nasopharynx	W.A.; Wiertsema et al., 2011	*hpd#3^−^, fucP^−^*
H19^c^	Nasopharynx	W.A.; Wiertsema et al., 2011	*hpd#3^−^, fucP^−^*
H34	Nasopharynx	W.A.; Wiertsema et al., 2011	*hpd#3^−^, fucP^−^*
H51^c^	Nasopharynx	W.A.; Wiertsema et al., 2011	*hpd#3^−^, fucP^−^*
H54^c^	Nasopharynx	W.A.; Wiertsema et al., 2011	*hpd#3^−^, fucP^−^*
H56^c^	Nasopharynx	W.A.; Wiertsema et al., 2011	*hpd#3^−^, fucP^−^*
H95	Nasopharynx^a^	W.A.; Wiertsema et al., 2011	*hpd#3^−^, fucP^−^*
H152	Nasopharynx	W.A.; Wiertsema et al., 2011	*hpd#3^−^, fucP^−^*
***H. influenzae—*****like (designated according to phylogenetic tree in** Price et al., 2015**)**			
H18	Nasopharynx	W.A.; Wiertsema et al., 2011	*hpd#3^+^, fucP^−^*
H40	Nasopharynx	W.A.; Wiertsema et al., 2011	*hpd#3^+^, fucP^−^*
H148	Middle ear	W.A.; Wiertsema et al., 2011	*hpd#3^+^, fucP^−^*
H180	Nasopharynx	W.A.; Wiertsema et al., 2011	*hpd#3^+^, fucP^−^*

a isolated from the same nasopharyngeal swab.

b The hpd#3 PCR is NTHi-specific (Wang et al., 2011) and therefore does not detect H. haemolyticus hpd.

c hpd-negative H. haemolyticus confirmed by PCR (Pickering et al., 2014a) and whole genome sequencing (Price et al., 2015).

ATCC, American Type Culture Collection; U.S.A, United States of America; W.A. Western Australia.

Bacteria were cultured from glycerol stocks, or resuspended lyophilised pellets, onto chocolate agar plates and incubated overnight at 37°C in 5% CO_2_. Inoculum for all subsequent experiments was harvested from agar plates as previously described for NTHi (Swords et al., 2000). Bacterial viability was measured as previously described (Kirkham et al., 2013).

### Epithelial cell culture and viability

All cell culture reagents were from Gibco, Life Technologies, Tullamarine, Victoria, Australia, unless otherwise stated. Cell cultures were maintained in 125 cm^3^ flasks (BD Biosciences, Macquarie Park, NSW, Australia) at 37°C, 5% CO_2._Immortalized Detroit 562 human pharyngeal carcinoma epithelial cells (ATCC® CCL-138) were cultured in Minimal Essential Media (MEM) with Earle's salts and supplemented with 10% heat inactivated fetal calf serum (Sigma-Aldrich), 1 mM sodium pyruvate, 2 mM L-glutamine, 1 × non-essential amino acids, and 100 U Penicillin/Streptomycin. Cells were weaned from antibiotics 24 h prior to suspension in 24-well tissue culture plates (Corning, Life Technologies). Immortalized A549 human lung carcinoma epithelial cells (ATCC® CCL-185) were cultured in RPMI 1640 (Invitrogen, Life Technologies) supplemented with 10% heat inactivated fetal calf serum. At confluence, flasks of each cell line were disrupted with 0.05% trypsin/EDTA, washed in 1 × PBS and resuspended in their respective culture media. Cells were counted with a haemocytometer and viability was determined by trypan blue staining. Then cells were seeded into 24-well plates at 1 × 10^5^/mL and grown to confluence for bacterial challenge.

### Bacterial association and invasion of respiratory epithelial cell lines

A standard gentamicin invasion assay was used to determine the ability of *H. haemolyticus* and NTHi to attach to and invade epithelial cells (Swords et al., 2000), with the following modifications. Monolayers of cells were challenged at a multiplicity of infection (MOI) of 10:1 bacteria:cells for 1, 3, or 24 h. At the 1 h gentamicin treatment step for evaluation of intracellular bacteria, media only was added to the cells being evaluated for total bacterial association for 1 h. 100 μg/mL of gentamicin was used instead of 50 μg/mL. All isolates were checked to be sensitive to 100 μg/mL gentamicin and resistant to 2% saponin as determined by viable count. Assays were conducted in triplicate and on at least three separate occasions. Supernatants from each triplicate well were pooled after bacterial challenge, filter-sterilized and stored at −80°C for measurement of inflammatory mediators.

### PBMC stimulation with bacteria

Adult PBMCs were collected from five healthy donors and processed and prepared as previously described (Kirkham et al., 2013). PBMCs were stimulated with thawed preparations of either NTHi86-028NP or *H. haemolyticus*33390 at 10:1 bacteria:cells. Cells in control wells were treated with either PBS (cells only; unstimulated), 1 ng/mL lipopolysaccharide (LPS, from *Escherichia coli* R515; Alexis Biochemicals, Sapphire Biosciences, NSW, Australia) or 1 μg/mL Staphylococcal enterotoxin B (SEB, from *Staphylococcus aureus*; Sigma Aldrich). At 24 h post-treatment, plates were centrifuged for 5 min at 200 g and supernatants were harvested. Triplicate wells were combined and stored at -80°C for measurement of inflammatory mediators.

### Cytokine and chemokine analysis

IL-6, IL-10, IFNγ, and TNFα levels (pg/mL) were measured in filter-sterilized supernatants from epithelial and PBMC cultures using the Bioplex 200 system (Bio-Rad Laboratories Inc., Hercules, CA, USA) and an in-house multiplex bead-based assay as previously described (Blyth et al., 2011). Measurement of IL-1β and IL-8 was added into this Bioplex assay using commercially available antibody pairs (Bioscientific, Kirrawee, NSW, Australia) and following validation to ensure that there was no cross reactivity with any of the other cytokines.

### Staining and flow cytometry

As NTHi is difficult to distinguish from *H. haemolyticus* by standard microbiological culture, we used the fluorescent dye Cell Trace Violet (CTV, excitation = 390 nm, emission = 445 nm; Invitrogen) to label NTHi86-028NP for experiments involving bacterial co-culture. Overnight growth of bacteria was harvested from chocolate agar plates and resuspended in cell culture media to an OD_600*nm*_ 0.2 (which is equivalent to ~10^8^ CFU/mL). Bacterial suspensions were incubated with 20 μM CTV for 5 min before pelleting at maximum speed in a benchtop centrifuge for 7 min. Harvested bacteria were resuspended in 10 mL of pre-warmed epithelial cell culture media, protected from light and incubated at 37°C for 10 min before use. The CTV stain did not affect the ability of NTHi86-028NP to attach to or invade A549 cells (data not shown). Flow cytometry was conducted on a Flow and cell cytometer Canto II device (BD Biosciences) that was calibrated using Cytometer Setup and Tracking beads (BD Biosciences). Forward scatter and side scatter were set to capture bacterial populations. Flow cytometry data was acquired using FACSDiva software and then analyzed using Flow Jo v7 software (FloJo LLC, Oregon, USA).

### Competitive colonization assay with detroit 562 cells

The optimal challenge dose of ~1 × 10^7^ CFU/mL for the association and invasion experiments was not possible for the competitive colonization experiment due to the high background fluorescence of unstained bacteria. We therefore used a higher challenge dose of 1 × 10^9^ CFU/mL CTV-NTHi (MOI 1000:1). NTHi86-028NP was prepared and stained as above however the bacterial suspension was concentrated to 1 × 10^9^ CFU/mL by harvesting 100 mL and resuspending in 10 mL prior to addition to epithelial cells. Overnight growth of *H. haemolyticus*33390 was harvested from chocolate agar plates and resuspended in cell culture media to an OD600 nm 0.2 (which is equivalent to ~10^8^ CFU/mL). D562 monolayers were treated with either 10-fold increasing doses (1 × 10^3^ to 1 × 10^7^ CFU/mL) of *H. haemolyticus*33390 or media alone for 1 h at 37°C, 5% CO_2_. Cells were then washed three times with PBS to remove unbound *H. haemolyticus* and challenged with 1 × 10^9^ CFU/mL CTV-labeled NTHi86-028NP in culture media for 1 h. Two control wells were treated with 1 × 10^9^ CFU/mL unstained NTHi to give background fluorescence levels. All wells were washed three times with 200 μL PBS to remove unbound NTHi and then 200 μL of PBS was aliquoted into each well. Each plate was then read immediately with an Enspire plate reader (Perkin Elmer, Massachusetts, USA).

### Transwell assay with A549 cells

High density 3 μM pore Transwell inserts (BD Biosciences) were seeded with 1 × 10^4^ A549 cells and cultured until confluent. As the high density inserts are opaque, it was not possible to gauge confluence of the cells on these inserts with a microscope. Therefore, two transparent low density 3 μM pore inserts were used to gauge confluence of A549 monolayers on the high density inserts. Transepithelial electrical resistance (TER) measurements were variable with A549 cells, therefore fluorescently conjugated dextrans RITC (Rhodamine B Isothiocyanate conjugated to 70 kDa dextran) and FITC (Fluoroscein Isothiocyanate conjugated to 3 kDa dextran; both Sigma) were used to determine the permeability of confluent epithelial cells seeded onto Transwells, as previously described (Kowapradit et al., 2010). The concentration of each fluorescent tracer was measured by the Enspire multi plate reader (FITC excitation = 485 nm, emission = 544 nm, RITC excitation = 520 nm, emission = 590 nm) using standard curves of known concentrations of RITC and FITC. Epithelial monolayers that were deemed confluent by microscopy were confirmed to be exclusive of passive bacterial transfer by the presence of 3 kDa but not 70 kDa fluorescent tracers in the lower compartment of the Transwell cultures.

When the low density inserts were deemed confluent with A549 cells, cells were counted from these two inserts and the average cell count was used to determine the bacterial challenge dose required for an MOI of 10:1. Media was removed from the inner chamber of the Transwell inserts and replaced with either media alone or media containing *H. haemolyticus* (MOI 10:1), prepared as described above for the bacterial association experiments. Treated cells were incubated at 37°C, 5% CO_2_ for 1 h. Inserts were then washed thoroughly with PBS, and CTV-stained NTHi (MOI of 10:1) was added to the inner chambers. Following a 3 h incubation at 37°C, 5% CO_2_, 100 μL of media from above and 100 μL of media from below the Transwells was removed for FACs analysis.

### Statistical analyses

Statistical analyses were conducted using GraphPad Prism version 5.02 (GraphPad Inc., California, USA) and *p* < 0.05 was considered statistically significant. Student's *t*-tests were used to compare log transformed bacterial viability and Mann-Whitney *U*-tests were used to compare epithelial viability and bacterial association and invasion of individual isolates with reference strains. Kruskal-Wallis test with Dunn's post-test analysis was used to compare intra-species association and invasion of epithelial cells, epithelial cytokine release and PBMC cytokine release. Student's *t*-tests were used to determine differences in bacterial fluorescence following different pre-treatments in competitive colonization assays.

## Results

### *H. haemolyticus* viability in cell culture media diminished significantly more than NTHi

**Figure 1:**
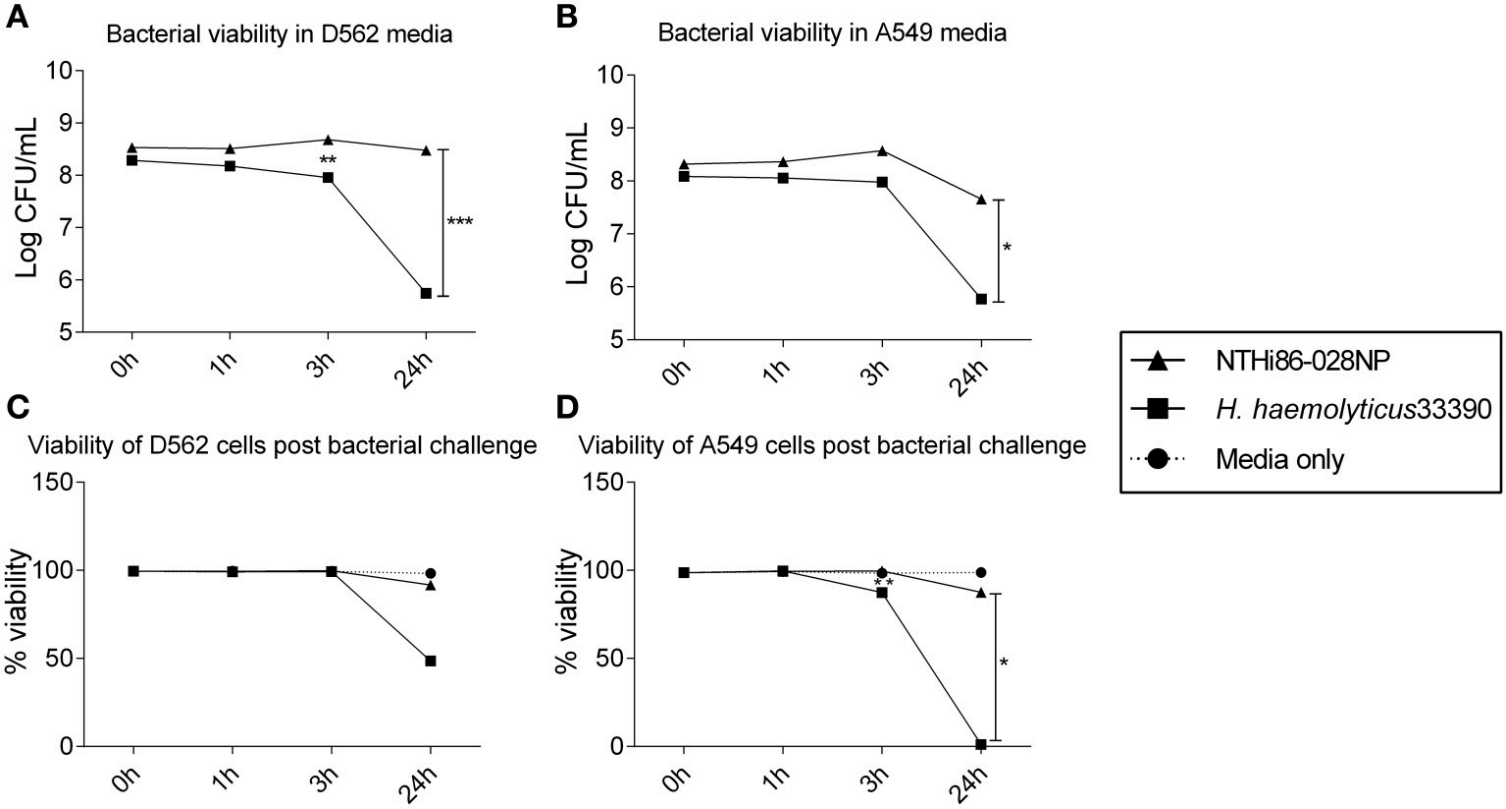
**Viability of respiratory epithelial cells co-cultured with *H. haemolyticus* and NTHi**. Viability of NTHi86-028NP and *H. haemolyticus*33390 in D562 **(A)** and A549 **(B)** cell culture media (no epithelial cells) over 24 h as determined by viable count.Viability of D562 **(C)** and A549 **(D)** respiratory epithelial cells incubated with media only (circles), *H. haemolyticus*33390 (squares), or NTHi86-028NP (triangles) over 24 h as determined by trypan blue counts. Student's *t*-tests were used to compare log transformed bacterial viability and Mann Whitney *U*-tests were used to compare post bacterial challenge epithelial cell viability, where *p < 0.05, **p < 0.01, ***p < 0.001.

The viability of reference strains *H. haemolyticus*33390 and NTHi86-028NP remained relatively stable in D562 and A549 media for the first 3 h of incubation. However, by 24 h the viability of *H. haemolyticus*33390 dropped significantly in D562 media (Figure 1A, *p* < 0.001) and A549 media (Figure 1B, *p* < 0.05) when compared to NTHi viability at 24 h.

### *H. haemolyticus* was cytotoxic, particularly to A549 epithelial cells

*H. haemolyticus*33390 challenge had no impact on D562 cell viability at 3 h, however the epithelial viability reduced to 50% by 24 h (Figure 1C). This cell death was even more pronounced with A549 cells, where viability following *H. haemolyticus*33390 challenge decreased from ~85% at 3 h to complete cell death at 24 h (Figure 1D). In contrast, NTHi had minimal impact on the viability of both cell lines over the same time period. Several *H. haemolyticus* isolates were also cytotoxic at 24 h (data not shown). In consideration of the viability results, a 3 h time point and D562 cells were chosen for further analysis of *H. haemolyticus* host cell association, invasion, and inflammatory properties.

### *H. haemolyticus* associated with epithelial cells but not as well as NTHi

**Figure 2:**
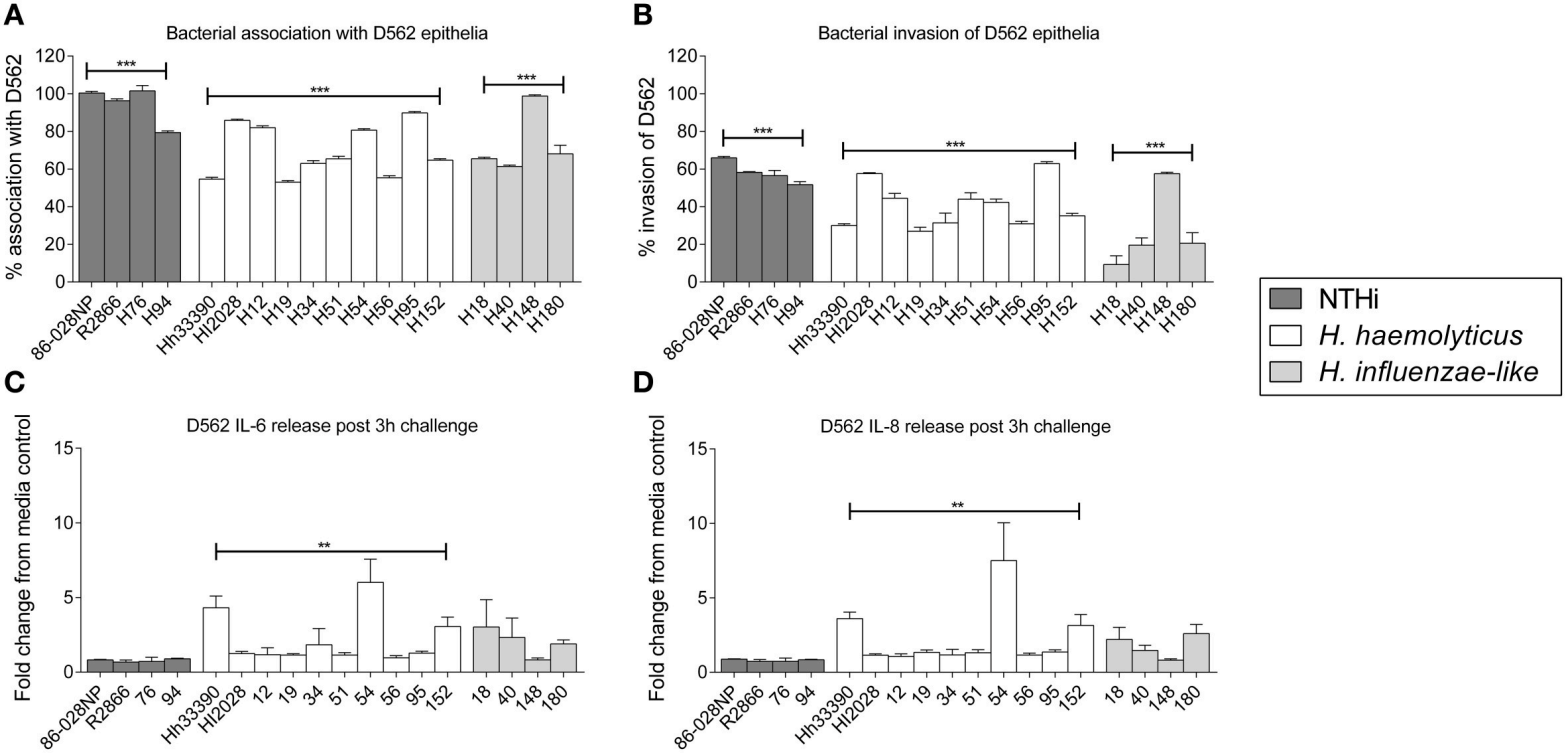
**Interactions of clinical strains of haemophili with respiratory epithelia**. Association **(A)** and invasion **(B)** of D562 epithelial monolayers with haemophilus reference strains and clinical isolates at 3 h. Level of IL-6 **(C)** and IL-8 **(D)** present in cell culture supernatant at 3 h post-bacterial challenge and presented as the fold change of cytokine in the supernatant from cells incubated with media only. Results are represented as the mean ± SEM of three separate experiments, each conducted in triplicate excluding R2866 which was conducted once in triplicate. Kruskal-Wallis and Dunn's post-test was used to compare intra-species variability where **p < 0.01; ***p < 0.001.

There was significant intra-species variability in the capacity of each haemophilus group to attach to and invade D562 cells (Figures 2A,B; *p* < 0.01). However, overall the NTHi isolates displayed a greater ability to associate with and invade D562 epithelia (range: association 76–122%, invasion 44–79%) compared with *H. haemolyticus* isolates (range: association 48–93%, invasion 0–70%) and *H. influenzae*-like isolates (range: association 49–101%, invasion 0–62%) (Figures 2A,B). The invasive *H. haemolyticus* isolate HI2028 and invasive *H. influenzae*-like middle ear isolate H148 had high levels of attachment (96 ± 1% and 99 ± 1%, respectively, Figure 2A) and invasion (58 ± 1% and 58 ± 1%, respectively, Figure 2B) that were comparable to the NTHi strains and significantly higher than the *H. haemolyticus*33390 reference strain (*p* < 0.01). However, this was not specific to the invasive *H. haemolyticus* strain as there were also carriage isolates of *H. haemolyticus* (H12, H54, H95) that had attachment and invasion properties similar to NTHi.

### Protein D is not essential for *H. haemolyticus* association with host cells

The absence of the protein D (*hpd*) gene in four clinical *H. haemolyticus* isolates (H19, H51, H54, and H56) was confirmed by whole genome sequencing (Price et al., 2015). Based on previous NTHi data, we hypothesized that the *H. haemolyticus hpd*-negative isolates would have significantly reduced association with host cells. This was not observed, with the four strains displaying significant variability in attachment (*p* < 0.01) and invasion (*p* < 0.01), Figures 2A,B. Compared with the *H. haemolyticus*33390 reference strain, two of the *hpd*-negative *H. haemolyticus* isolates (H19, H56) had similar association properties (*p* = 0.75 and *p* = 0.44, respectively) and 2 (H51, H54) had significantly higher association properties (*p* < 0.01 and *p* < 0.01, respectively).

### Some *H. haemolyticus* isolates induced high pro-inflammatory responses from epithelial cells whereas NTHi did not

There was significant variation in epithelial release of IL6 (*p* < 0.01, Figure 2C) and IL-8 (*p* < 0.01, Figure 2D) in response to challenge with the 10 *H. haemolyticus* isolates investigated. In contrast, there was negligible variability in the IL-6 and IL-8 cellular responses to the different NTHi strains (*p* = 0.35 and *p* = 0.77, respectively). The D562 IL-6 and IL-8 responses to NTHi challenge were lower than those produced by unstimulated D562 cells. The invasive *H. haemolyticus* HI2028 isolate behaved like NTHi in terms of eliciting low levels of IL-6 and IL-8 compared with other *H. haemolyticus* strains but this was not unique to the invasive strain. Similarly, the *H. influenzae*-like middle ear isolate H148 lacked inflammatory properties compared to the other *H. influenzae*-like strains, which matched the increased association and invasion profile for the H148 strain. Strain H54, an *hpd*-negative isolate, was the most immunostimulatory of all strains assessed, but this high IL-6 and IL-8 response was not observed for the other *hpd*-negative isolates (H19, H51, and H56). IFNγ, TNFα, IL-10, and IL-1β measurements were below the limit of detection in culture supernatant at the 3 h time-point (data not shown).

### NTHi-treatment of PBMCs elicited IFNγ production whereas *H. haemolyticus*-treatment did not

**Figure 3:**
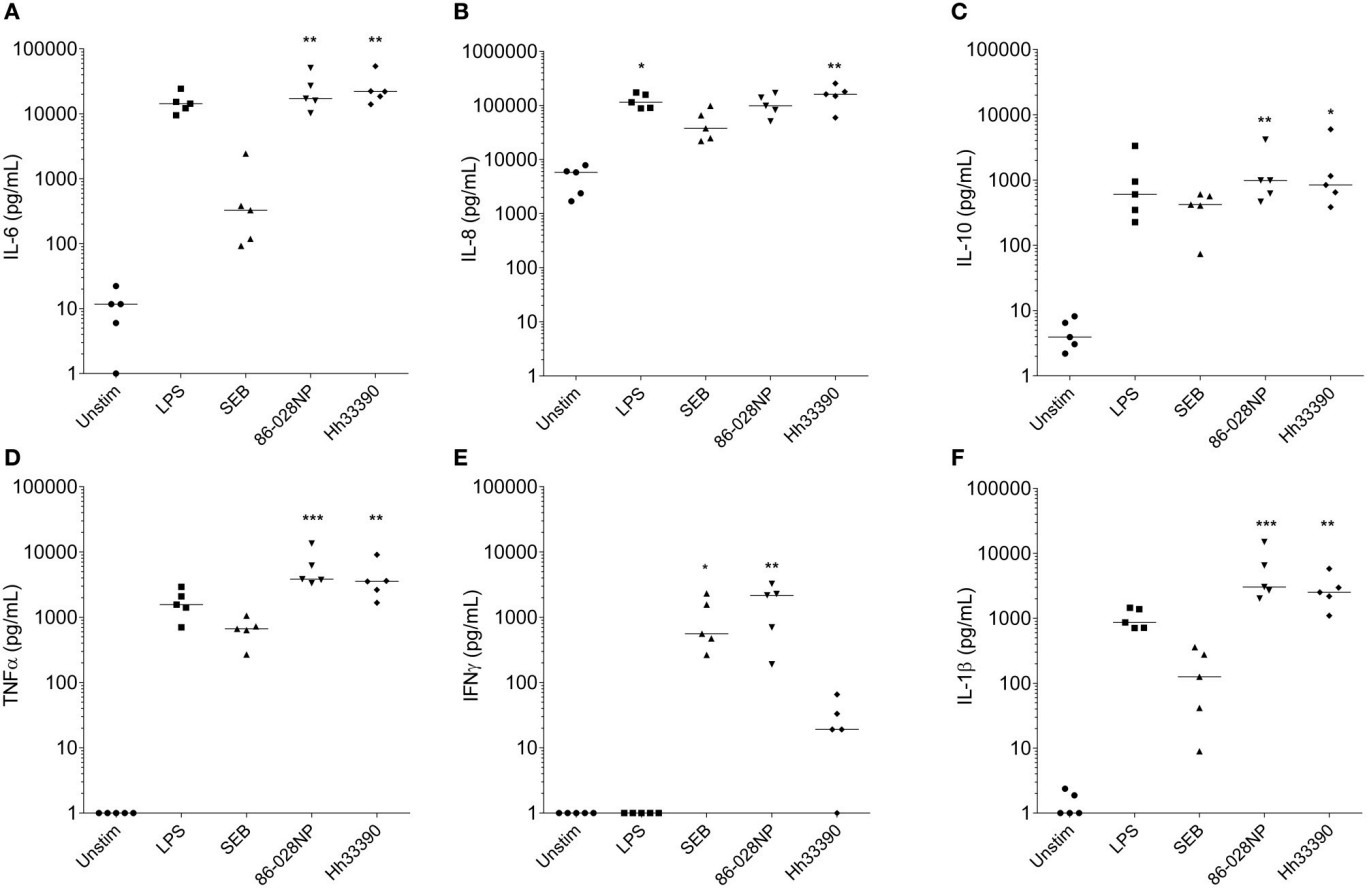
**Inflammatory mediator release (pg/mL) from PBMCs at 24 h post-treatment with NTHi or *H. haemolyticus***. Concentration of inflammatory mediators IL-6 **(A)**, IL-8 **(B)**, IL-10 **(C)**, TNFα **(D)**, INFγ **(E)**, and IL-1β **(F)** following treatment of adult PBMCs with either media alone (unstim), lipopolysaccharide (LPS), Staphylococcal enterotoxin B (SEB), NTHi86-028NP, or *H. haemolyticus*33390. The horizontal bars depict the median analyte level for each treatment group (*n* = 5). *p < 0.05, **p < 0.01; ***p < 0.001 when compared with unstimulated cells using a Kruskal-Wallis and Dunn's post-test.

Extracellular levels of IL-6, IL-10, TNFα, and IL-1β were significantly increased following 24 h stimulation of PBMCs with either NTHi86-028NP (*p* < 0.01) or *H. haemolyticus*33390 (*p* < 0.05) when compared to unstimulated cells (Figure 3). *H. haemolyticus* and LPS induced significant release of IL-8 from PBMCs compared with unstimulated cells (*p* < 0.01 and *p* < 0.05, respectively). IL-8 production from PBMCs was also elevated in response to NTHi but this was not significant. In contrast, NTHi and SEB induced significant release of IFNγ from PBMCs (*p* < 0.01 and *p* < 0.05, respectively) whereas *H. haemolyticus* did not. There was minimal IFNγ released from PBMCs treated with *H. haemolyticus* (mean 28 ± 10 pg/mL) compared with NTHi (mean 1718 ± 560 pg/mL).

### Pre-treatment of epithelial cells with *H. haemolyticus* inhibited NTHi association and transcytosis

**Figure 4:**
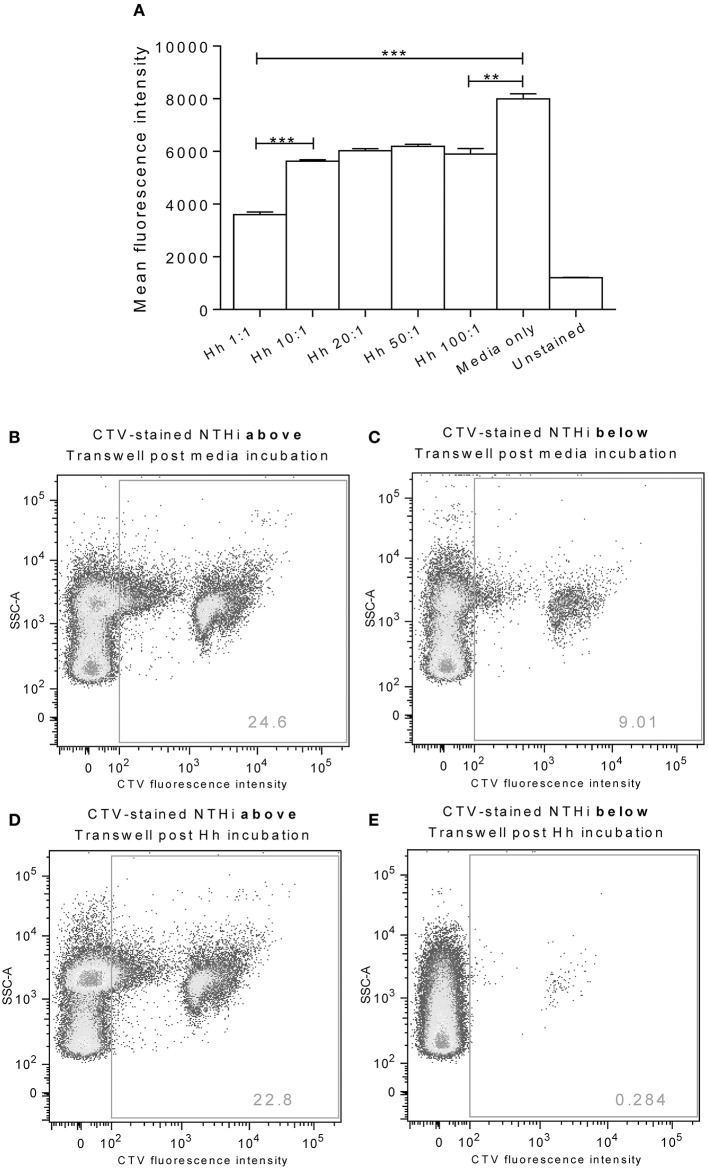
**Pre-colonization of epithelia with *H. haemolyticus* impacts subsequent NTHi interactions**. Monolayers of D562 cells were challenged with increasing doses of *H. haemolyticus*33390 or media for 1 h, washed thoroughly and then challenged with 1000:1 CTV-stained NTHi86-028NP **(A)**. At 1 h post NTHi-infection, wells were thoroughly washed, and fluorescence was measured to identify attached NTHi. Results are represented as mean ± SEM of three technical repeats. Student's unpaired *t*-test was used to compare between treatments where **p < 0.01, ***p < 0.001. A549 monolayers grown on Transwells were incubated with media **(B,C)** or 10:1 *H. haemolyticus*33390 **(D,E)** for 1 h. Monolayers were then washed with media, challenged with CTV-stained NTHi (MOI 10:1) and incubated at 37°C 5% CO_2_ for a further 3 h. Pre-colonization with *H. haemolyticus* (MOI 10:1) reduced the proportion of CTV-stained NTHi detected in the lower compartment of the Transwell (**E**, 0.3%) compared with pre-incubation with media alone (**C**, 9.0%).

Incubation of D562 cells for 1 h with increasing doses of *H. haemolyticus*33390 resulted in a significant reduction in the subsequent association of CTV-labeled NTHi86-028NP (Figure 4A). The greatest reduction of NTHi association was observed when D562 cells were pre-incubated with the lowest *H. haemolyticus* dose (MOI 1:1, *p* < 0.0001).

A Transwell chamber assay was used to examine the ability of *H. haemolyticus* to block NTHi transcytosis through a monolayer of A549 epithelial cells (Figures 4B–E). We observed that pre-treatment of Transwell cultured A549 cells with *H. haemolyticus*33390 (MOI 10:1, ~1 × 10^7^ CFU/mL) reduced NTHi transcytosis by 30-fold (Figure 4E) when compared with cells treated with NTHi alone (Figure 4C); 0.3 vs. 9% fluorescence.

## Discussion

To our knowledge, this is the first study to assess interactions of *H. haemolyticus* with host cells. We have developed *in vitro* models that can be used to further investigate the interaction of *H. haemolyticus* with the host. The loss in *H. haemolyticus* viability in cell culture media over 24 h indicates bacterial death but the mechanisms behind this (i.e., autolysis or allolysis) have not been determined. We observed that, in contrast to NTHi, *H. haemolyticus* is cytotoxic to immortalized nasopharyngeal and bronchoalveolar epithelial cell lines over a 24 h incubation period. This was surprising given the predominantly commensal nature of *H. haemolyticus*. As *H. haemolyticus* does not grow well in liquid media, it is possible that bacterial death results in release of cytotoxic components over 24 h. Indeed, filtered cell culture media from some *H. haemolyticus* strains incubated for 24 h with D562 cells was cytotoxic to the immortalized epithelial cell lines (data not shown), suggesting that a soluble cytotoxic component is released into the cell culture media. It is possible that some *H. haemolyticus* strains express a toxin that is not expressed by NTHi. Indeed, *H. ducreyi* is known to secrete cytolethal distending toxin, which arrests the epithelial cell cycle (Gargi et al., 2013). Comparative genomics has revealed putative *H. haemolyticus* toxins that are under further investigation in our laboratory. It is also possible that our method for preparing inoculum (harvesting bacteria from agar plates rather than liquid culture) results in a high proportion of dead bacteria that are not accounted for by viability counting. These dead bacteria may lead to an increase in toxic load to the epithelial cells, possibly from lipooligosaccharide (LOS) or other toxins. However, the *H. haemolyticus* and NTHi inocula were prepared in the same manner and the NTHi preparation was not cytotoxic. Differences in the phosphorylcholine (ChoP) decoration of LOS between *H. haemolyticus* and NTHi have been identified and have been suggested to provide an explanation for NTHi pathogenesis through promotion of bacterial adherence and invasion of host cells (McCrea et al., 2010b; Post et al., 2016). Detailed comparison of LOS structures from nine *H. haemolyticus* and six NTHi strains found that only 4/9 (44%) of the *H. haemolyticus* strains tested expressed ChoP decorated LOS in comparison with 5/6 of NTHi strains (Post et al., 2016). Interestingly, we found that a similar proportion of the *H. haemolyticus* strains in our study (4/10) had better attachment to epithelial cells that was similar to the NTHi strains (>80% association of challenge inoculum; Figure 1). Whether ChoP decoration of LOS facilitates *H. haemolyticus* association with epithelial cells remains to be determined. It is important to understand the basis of the observed cytotoxicity and intra-species variation if *H. haemolyticus* is to be further assessed as a potential probiotic. Identification of virulence genes and recombination hotspots in *H. haemolyticus* will highlight regions that will require attenuation or natural selection.

Our hypothesis was that *H. haemolyticus* would not associate with and/or invade epithelial cells in the same capacity as NTHi. All 10 *H. haemolyticus* strains investigated were able to infect cells within 3 h (with most association and invasion occurring in the first hour). Although *H. haemolyticus* was hypothesized to have reduced cell association and invasion overall compared with NTHi, there was considerable intra-species variation. Such strain to strain variation was expected, given how closely related *H. haemolyticus* is to the genetically heterologous NTHi (Erwin and Smith, 2007; Price et al., 2015) and the ability for recombination between these species (Sondergaard et al., 2014). The greatest difference between *H. haemolyticus* and NTHi attachment and invasion was evident between the reference strains, highlighting the necessity for assessing multiple strains within a species to gain an understanding of strain diversity.

Protein D, the NTHi vaccine antigen in PHiD-CV, has been demonstrated to be important for adherence of NTHi to airway epithelial cells (Johnson et al., 2011). In contrast, Protein D does not appear to be required for *H. haemolyticus* association with host cells, with the four different Protein D-negative strains exhibiting higher or equal attachment to and invasion of cells than Protein D-positive strains. A protein D knockout in *H. haemolyticus* would need to be constructed and compared with the parent strain to confirm that Protein D is not essential for *H. haemolyticus* adherence to epithelial cells. The identification of naturally-occurring Protein D-negative NTHi carriage isolates (Smith-Vaughan et al., 2014) also suggests that this vaccine antigen is not required for NTHi colonization of the host.

The invasive *H. haemolyticus* isolate HI2028 (Morton et al., 2012) interacted with the host cells in a similar manner to the NTHi isolates, with comparable cell association, invasion, and low inflammatory responses. However, this was not exclusive to HI2028 and other non-invasive *H. haemolyticus* carriage isolates displayed similar host interactions. Comparative genomics of the virulome for HI2028 vs. other haemophili may provide insight into whether there are specific genes (possibly acquired from NTHi) that are required for *H. haemolyticus* to become invasive.

We also assessed the host-cell interactions of four strains that belong to a recently described clade of “*H. influenzae*-like” haemophili (Price et al., 2015). These strains are identified as NTHi by the #*hpd*3 PCR (Wang et al., 2011) but are lacking the fucose operon that is used to identify NTHi with the multi-locus sequence typing scheme (http://haemophilus.mlst.net/; de Gier et al., 2015; Price et al., 2015). Overall, the *H. influenzae*-like haemophili were more similar in their host cell interactions to the *H. haemolyticus* than the NTHi strains assessed in this study, with lower attachment and invasion of epithelial cells and higher immunostimulatory properties. Interestingly, the one *H. influenzae*-like strain that displayed NTHi-like properties (H148) was isolated from the middle ear effusion of a child with recurrent acute OM whereas the other *H. influenzae*-like strains were nasopharyngeal isolates. The intra- and inter-species variability in host interactions that were observed in this study highlights the complexity of species definition in the genomic era. Awareness of the *H. influenzae*-like cluster and further analysis of the phenotypic traits of such strains is required to assist in resolving Haemophilus speciation.

*H. haemolyticus* was overall more immunostimulatory to epithelial cells than NTHi, with negligible release of IL-8 and IL-6 at 3 h post-NTHi stimulation. This suggests that NTHi is able to down-regulate or evade the initial immune responses in this model. The majority of the literature on host epithelial responses to NTHi are either with bacterial lysates or purified bacterial components rather than live NTHi. In support of our findings, assessment of A549 epithelial cell inflammatory responses to NTHi found that live NTHi did not stimulate activation of the IL-8 promoter, whereas treatment of the A549 cells with a soluble cytoplasmic fraction of NTHi induced robust IL-8 promoter activation within 4 h (Wang et al., 2003). This is further supported in a study describing NTHi isolates that did not stimulate mRNA expression of any cytokine or chemokine in human middle ear epithelial cells (Tong et al., 2001). The mechanisms for NTHi evasion of the host epithelial inflammatory response are not fully understood but it appears that some strains of *H. haemolyticus* also possess this property. Interestingly, gut commensals have been shown to either attenuate host production of inflammatory cytokines (Neish et al., 2000) or elicit a local inflammatory response (Chervonsky, 2012) to regulate colonization. It is possible that *H. haemolyticus* may use similar methods for regulating colonization of the respiratory tract. The host response to polymicrobial challenge has not been taken into consideration in our study and the synergistic effect of NTHi and *H. haemolyticus*, as well as other respiratory flora, warrants investigation. For example, there is an increase in IL-8 production from A549 and D562 cells when they are simultaneously challenged with NTHi and *Streptococcus pneumoniae*, compared with single species challenge (Ratner et al., 2005). Understanding regulation of microbial colonization at the epithelial surface may lead to the development of strategies to promote healthy colonization of the human respiratory tract and ultimately to the prevention of respiratory tract infections.

Further differences in cell-mediated responses to NTHi and *H. haemolyticus* were observed with PBMCs, where NTHi86-028NP challenge released high levels of IFNγ but *H. haemolyticus*33390 did not. IFNγ is critical in clearing intracellular bacterial infections such as *Listeria monocytogenes* and *Salmonella typhimurium* (Suzue et al., 2003). Intracellular survival provides a reservoir of bacteria for reinfection and is thought to contribute to the recurrent nature of diseases such as OM and exacerbations in bronchiectasis patients (Coates et al., 2008; King et al., 2008). We have shown that NTHi can persist intracellularly in the middle ear of children with recurrent OM (Thornton et al., 2011). Although a specific role for IFNγ in clearing NTHi infection has not been identified, PBMCs from children with chronic suppurative lung disease (Pizzutto et al., 2015) and adults with bronchiectasis (King et al., 2008) have been shown to have impaired IFNγ production in response to NTHi challenge. The lack of IFNγ response from *H. haemolyticus* treated PBMCs may suggest that *H. haemolyticus* cannot survive inside PBMCs or it may just be that the *H. haemolyticus* has died in cell culture media (as observed with the epithelial cell culture media by 24 h). Indeed, we have previously shown that live preparations of NTHi (instead of heat-killed or ethanol-killed) are required for IFNγ production from PBMCs (Kirkham et al., 2013). A limitation of this study is that bacterial viability was not assessed due to the difficulty in distinguishing a loss in bacterial viability from cell-mediated killing. There was significantly more IL-8 production from *H. haemolyticus* treated PBMCs compared with NTHi-challenge, whether this is due to release of a soluble factor from live or dead *H. haemolyticus* remains to be determined.

Pre-treatment of D562 monolayers with all doses of *H. haemolyticus* reduced the proportion of NTHi that were subsequently able to associate with the epithelial cells. The lowest dose of *H. haemolyticus* (MOI 1:1) resulted in the greatest reduction of NTHi association, suggesting that the inhibitory effect of *H. haemolyticus* on NTHi attachment may be more than just competition for binding sites. These data, together with the immunostimulatory capacity of *H. haemolyticus*, suggest that immune activation may have a role in prevention of NTHi attachment and subsequent invasion. It is important to note that NTHi attachment was inhibited by *H. haemolyticus*, despite needing to use a high NTHi challenge dose (1 × 10^9^ CFU/mL) to detect fluorescence above background levels for unstained NTHi. In attempts to improve sensitivity for assessment of whether *H. haemolyticus* could block NTHi association and infection, a Transwell assay was investigated. We observed that pre-colonization of A549 monolayers with *H. haemolyticus* reduced the proportion of NTHi that could translocate through the epithelial cells. Whilst this result was also promising, the Transwell model we employed has limitations and others have suggested A549 cells do not express functional tight junction proteins when grown as a monolayer (Balis et al., 1984; Winton et al., 1998). This is likely to have an impact on the integrity of the epithelial barrier, and suggests that more complex models are required to investigate the potential of *H. haemolyticus* to prevent NTHi association and invasion.

In summary, we have demonstrated that the interaction of *H. haemolyticus* with the host is fundamentally different to NTHi, despite their genetic similarity and ability to recombine. We have indicated that the *H. influenzae*-like cluster of NTHi strains interact with the host in a more similar manner to *H. haemolyticus* than NTHi, in terms of epithelial association and cytokine responses. Our data suggest that *H. haemolyticus* may block NTHi colonization, providing the foundation for further assessment of *H. haemolyticus* as a bacterial therapy to prevent NTHi diseases.

## Authors contributions

LK and PR conceived the project. LK, JP, and RT designed the experiments. JP, AP, KC, and CG executed the experiments and all authors contributed to data interpretation. JP and LK prepared the manuscript and all authors provided critical review.

## Funding

This work was funded by the Australian National Health and Medical Research Council (NHMRC; project grants 1011172 and 1086580) and the Western Australian Department of Health and Channel 7 Telethon Trust.

### Conflict of interest statement

The authors declare that the research was conducted in the absence of any commercial or financial relationships that could be construed as a potential conflict of interest.

## Abbreviations

A549: human lung carcinoma epithelial cells
CFU: colony forming units
ChoP: phosphorylcholine
CTV: cell trace violet
D562: Detroit 562 human pharyngeal carcinoma epithelial cells
IFN: interferon
IL: interleukin
LOS: lipooligosaccharide
LPS: lipopolysaccharide
NTHi: nontypeable *Haemophilus influenzae*
OM: otitis media
PBMCs: peripheral blood mononuclear cells
RT: room temperature
SEB: Staphylococcus enterotoxin B
SEM: standard error of the mean
TNF: tumor necrosis factor.
